# Approach to the sonographic evaluation of fetal ventriculomegaly at 11 to 14 weeks gestation

**DOI:** 10.1186/s12884-016-0797-z

**Published:** 2016-01-12

**Authors:** Gwendolin Manegold-Brauer, Anton Oseledchyk, Anne Floeck, Christoph Berg, Ulrich Gembruch, Annegret Geipel

**Affiliations:** Department of Obstetrics and Prenatal Medicine, University of Bonn, Sigmund-Freud-Str. 25, Bonn, Germany; Department of Prenatal Medicine and Gynecologic Ultrasound, University of Basel, Basel, Switzerland; Department of Obstetrics and Gynecology, Division of Prenatal Medicine and Gynecologic Ultrasound, University of Cologne, Cologne, Germany

**Keywords:** First trimester, Hydrocephalus, Prenatal ultrasound, Ventriculomegaly

## Abstract

**Background:**

The aim of the study was to report the prevalence and associated findings of fetal ventriculomegaly between 11 + 0 and 13 + 6 gestational weeks and to evaluate a sonographic approach to classify first trimester ventriculomegaly in the standard axial plane used for biparietal diameter (BPD) measurement.

**Methods:**

The ratio between choroid plexus and lateral ventricle diameter (PDVDR), between the choroid plexus and lateral ventricle length (PLVLR) and between the choroid plexus and lateral ventricle area (PAVAR) were calculated from stored 2D images of the axial head plane in 100 normal fetuses and 17 fetuses with ventriculomegaly.

**Results:**

The PDVDR, the PLVLR and the PAVAR were below the 5^th^ percentile in 82.4 %, 94.1 % and 94.1 % of the cases with ventriculomegaly. Ventriculomegaly was isolated in 29.4 % and associated with further anomalies in 70.6 % at the initial evaluation. The mean PLVLR in euploid compared to aneuploid fetuses was significantly lower (0.40 versus 0.53 (*p* = 0.0332)).

**Conclusions:**

The measurements of PDVDR, PLVLR and PAVAR are helpful to objectify ventriculomegaly at 11–14 gestational weeks. The PLVLR and PAVAR were superior to PDVDR, since there seems to be rather shrinkage of the choroid plexus than an increased width of the lateral ventricles in the first trimester.

## Background

In recent years, the 11–14 weeks scan expanded from a scan mainly exploring gestational age by measurement of crown-rump-length and assessing the risk of aneuploidy by measuring nuchal translucency (NT), to an early anomaly scan that includes a checklist for the assessment of the fetal anatomy similar to the 20 weeks scan. Although it is still not a part of routine screening, fetal structural abnormalities are increasingly detected during the first trimester [1]. A wide range of fetal anomalies have been reported [1–3]. The detection rate of major anomalies in the first trimester is higher in fetuses with increased NT as the awareness of the examiner is presumably higher. However, many malformations occur in fetuses without a coexistent increased NT [4, 5].

While first trimester fetal echocardiography has been reported in large studies [1–4, 6, 7], the current interest of several centers has been focused on first trimester brain anatomy [8–11]. Until pilot studies on posterior brain abnormalities in the midsagittal view demonstrated that the detection of spina bifida and Dandy–Walker malformation was feasible at 11–14 weeks [12–14], the detection rates of ventriculomegaly, agenesis of the corpus callosum and Dandy–Walker malformation were reported below 5 % in the first trimester [1]. Visualization of the choroid plexus within the lateral ventricles, the so–called “butterfly” sign in the transverse view of the fetal head, is one of the standard sections obtained. This view has been described helpful for the diagnosis of first trimester holoprosencephaly [11]. Further, the transverse diameter of the lateral ventricles and the relation to the area of the choroid plexus can be examined in this view. In fetuses with open spina bifida, the area of the lateral ventricles has been reported significantly decreased compared with controls [9].

Compared to the second trimester, ventriculomegaly is not well defined in the first trimester. This might be due to the rare occurrence in early pregnancy. Syngelaki et al. reported only 1 of 11 cases of ventriculomegaly evident at the first trimester examination [1]. In other studies, first trimester detection of ventriculomegaly was reported to be up to 16 % [1]. As in the second trimester, there might be an association to chromosomal disorders, genetic syndromes, brain malformations, vascular events or infection [15].

In two recent studies, using transvaginally obtained three-dimensional ultrasound brain volumes, the ratio between choroid plexus and lateral ventricle area was calculated retrospectively and evaluated for chromosomal abnormalities and spina bifida [9, 10]. However, in daily routine practice the majority of scans is performed in a two-dimensional (2D) transabdominal setting. We therefore aimed to evaluate an easier approach to classify first trimester ventriculomegaly in the standard axial plane used for biparietal diameter (BPD) measurement. Furthermore, we report the prevalence and associated findings of ventriculomegaly between 11 + 0 and 13 + 6 gestational weeks.

## Methods

This was a retrospective study including all pregnancies that were diagnosed with ventriculomegaly during first trimester examination (11 + 0 to 13 + 6 weeks of gestation). Between January 2004 and September 2014, 9167 women received a detailed first trimester anomaly scan. Fetal examination was performed transabdominally in analogy to the 20 weeks anomaly scan. A mid-sagittal view of the fetal profile was obtained to evaluate the NT and the nasal bone. Further, the examination of the fetal head included the visualization of the axial plane to measure fetal BPD. In this view, the midline echo of the falx cerebri and both plexi choroidei, forming the typical “butterfly sign“ were visualized and documented.

The viewpoint database search for “ventriculomegaly” and “hydrocephalus” identified 17 cases. The diagnostic evaluation of ventriculomegaly was subjectively classified with a 2D transabdominal and/or transvaginal ultrasound approach by one of the three senior level III fetal medicine specialists (AG; CB; UG) at our unit. In cases of suspected ventriculomegaly, a detailed anatomic evaluation of the fetus was performed including fetal neurosonography. This comprised the examination of the fetal head at a large magnification in several axial and saggital planes, inspecting the lateral ventricles, the third ventricle, the aqueduct of Sylvius, the brain stem including visualization of the intracranial translucency and the fourth ventricle. Basically the examiners used pattern recognition and their extensive experience in fetal neurosonography as the primary tool for establishing the diagnosis. In their experience often the initial less experienced examiner had difficulties in obtaining the butterfly sign. Factors that were crucial for the diagnosis were the impression of enlarged ventricular fluid in the brain compared to the size of the plexi choroidei.

The study group stems from a high risk collective in a tertiary level referral center of prenatal medicine. The patients are referred to our unit for various reasons. Common referrals for the examination were advanced maternal age, suspected fetal anomalies, a family history of genetic disorders and aneuploidies or anomalies in previous pregnancies. Cases of alobar holoprosencephaly were not included.

**Fig. 1:**
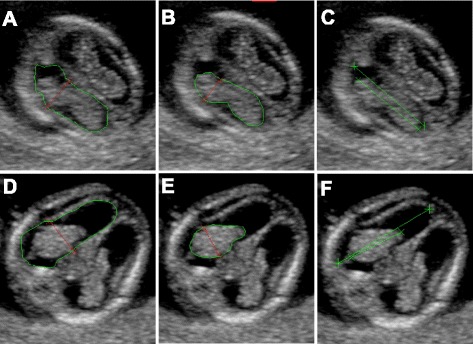
2D transverse view of the fetal brain demonstrating the measurements in a normal fetus (**a**-**c**) and in a fetus with ventriculomegaly (**d**-**f**): lateral ventricle diameter and lateral ventricle area (**a**, **d**); choroid plexus diameter and choroid plexus area (**b**; **e**); the choroid plexus length and lateral ventricle length (**c**;** f**)

In cases with documented ventriculomegaly the area, length and the transverse diameter of the choroid plexus and lateral ventricle were obtained retrospectively, using the metering tools of the PIA fetal database (Fig. 1). The ratio between choroid plexus and lateral ventricle diameter (PDVDR), the ratio between choroid plexus and lateral ventricle length (PLVLR) and the ratio between the choroid plexus and lateral ventricle area (PAVAR) were calculated.

To create reference ranges for normal PDVDR, PLVLR and PAVAR at 11–13 gestational weeks, 100 stored 2D images of the axial view of the head were randomly chosen from fetuses presenting in 2013 with known normal outcome. A normal outcome was defined as an unremarkable postnatal pediatric assessment in the first week after delivery. To assess the intraclass correlation, 50 images were independently measured by two trained examiners (GM; AF).

The responsible ethics committee (Ethikkommission an der Medizinischen Fakultät der Rheinischen Friedrich-Wilhelms-Universität Bonn, Germany) was informed about this study and confirmed that ethical approval is not required for this anonymous retrospective data analysis according to the national guidelines (No. 243/15).

### Statistical analysis

In normal fetuses regression analysis was used to examine whether each measurement changed with BPD and whether the relationship was linear or non-linear. Inspection of the residuals found no discrepancy from linearity.

The distribution of measurements of PDVDR, PLVLR and PAVAR were found to be normal around the linear regression line (Shapiro-Wilk normality test).

A 90 % prediction interval (5^th^, 50^th^ and 95^th^ percentiles) around the regression line was estimated based on the relationship to BPD. Inter-observer variability was assessed with intraclass correlation coefficients (ICC) and with Bland-Altman plots.

The statistical software R version 3.1.1 was used for data analysis.

## Results

The prevalence of ventriculomegaly between 11–14 gestational weeks in this tertiary referral center was 0.2 % (17/9167).

Ventriculomegaly was isolated in 5 cases (29.4 %) and associated with further sonographic anomalies in 12 cases (70.6 %) at the initial evaluation. There were two central nervous system abnormalities in euploid fetuses diagnosed at a follow-up scan, one with isolated agenesis of the corpus callosum and one with Dandy-Walker malformation (Cases No.4 and 5 , Table 1). There was no follow-up in the remaining 3 cases with suspected isolated ventriculomegaly, as termination of pregnancy was requested by the parents. In one case (No. 6) with Megacisterna magna as additional anomaly, Dandy-Walker malformation was suspected, but was finally confirmed at 20 weeks.

**Table 1:** Abnormal ultrasound findings, karyotype and outcome in cases with first trimester ventriculomegaly

No.	GA	NT (mm)	Additional abnormal ultrasound findings	Karyotype	Outcome
1	12 + 5	1.9	None	46 XY	1st trimester TOP
2	13 + 2	2.7	None	46 XX	1st trimester TOP
3	13 + 1	1.7	None	unknown	1st trimester TOP
4	12 + 6	1.4	None at initial evaluation, Agenesis of the corpus callosum (diagnosed at 20 weeks)	46 XY	2nd trimester TOP
5	13 + 2	2.3	None at initial evaluation, Dandy-Walker malformation (diagnosed at 15 weeks)	46 XX	Live born 32 + 5
6	12 + 5	1.7	Megacisterna magna (diagnosed with Dandy-Walker malformation at 20 weeks)	46 XY	2nd trimester TOP
7	13 + 1	1.5	Encephalocele	POMT1-mutation (Walker-Warburg syndrome)	1st trimester TOP
8	13 + 3	1.5	Occipital meningocele, megacisterna magna (diagnosed with Dandy-Walker malformation, abnormal fetal profile and short limbs at 16 weeks)	46 XY	2nd trimester TOP
9	12 + 0	3.0	Spina bifida, tetralogy of Fallot, right aortic arch, bilateral hydronephrosis, hexadactyly	46 XY	1st trimester TOP
10	12 + 3	1.6	Semilobar holoprosencephaly, median facial cleft, hexadactyly	46 XX	1st trimester TOP
11	12 + 4	3.0	Hexadactyly, bilateral cleft, absent nasal bone, VSD, common arterial trunc	Trisomy 13	1st trimester TOP
12	13 + 1	3.7	Bilateral cleft, hexadactyly, omphalocele, single umbilical artery	Trisomy 13	1st trimester TOP
13	12 + 6	2.8	Iniencephaly, VSD, absent pulmonary valve syndrome, wrist drop, limb fixation	Trisomy 18	1st trimester TOP
14	13 + 5	5.1	Artrio-ventricular septal defect	Trisomy 21	1st trimester TOP
15	13 + 1	2.8	Semilobar holoprosencephaly, median cleft, unilateral anopthalmy	unknown	1st trimester TOP
16	12 + 4	4.0	Spina bifida, omphalocele, cardiac anomaly	unknown	1st trimester TOP
17	12 + 1	7.2	Megacisterna magna, bilateral cleft, bilateral pyelectasia	unknown	1st trimester TOP

*GA* Gestational age at diagnosis, *NT* Nuchal translucency, *VSD* Ventricular septal defect, *TOP* Termination of pregnancy

Karyotyping was performed in 13 cases and 30.7 % (4/13) had an aneuploidy. The case of Walker–Warburg syndrome was a familial recurrence. NT was increased in 52.9 % (9/17), including all cases with chromosomal abnormalities (Table 1).

**Fig. 2:**
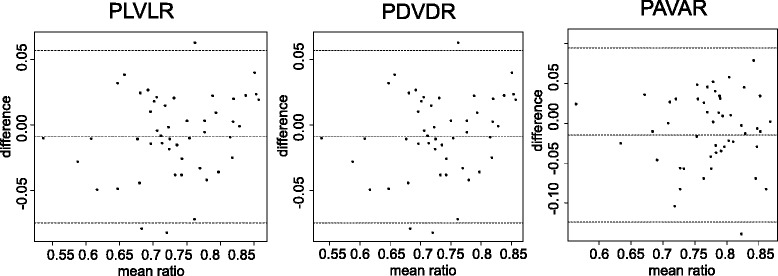
Bland-Altman plots showing the distribution oft he measurements performed by the two observers. Dotted lines mark mean and 95 % confidence limits. PDVDR: Choroid plexus to lateral ventricle diameter ratio; PLVLR: choroid plexus to lateral ventricle length ratio; PAVAR: choroid plexus to lateral ventricle area ratio

The BPD was within the normal range in 70.6 % (12/17) and above the 95^th^ percentile in 29.4 % (5/17) of the cases. Figure 1 illustrates the measurement of the three evaluated ratios. Expected choroid plexus diameter to lateral ventricle diameter ratio (PDVDR) was - 0.006167 x BPD + 0.870983; expected choroid plexus length to lateral ventricle length ratio (PLVLR) = − 0.009623 x BPD + 0.961766; expected choroid plexus area to lateral ventricle area ratio (PAVAR) = −0.01186 x BPD + 0.84078. The intraclass correlation coefficient between the two observers was 0.89 (CI 0.79-0.94) for PLVLR, 0.63 (CI 0.34-0.81) for PDVDR and 0.82 (CI 0.69-0.90) for PAVAR. Bland-Altman plots showed that there was no systematic relationship of mean and difference between the two observers. The limits of agreement were between −0.1 and 0.1 and therefore discrepancy was less then 10 % (Fig. 2).

**Fig. 3:**
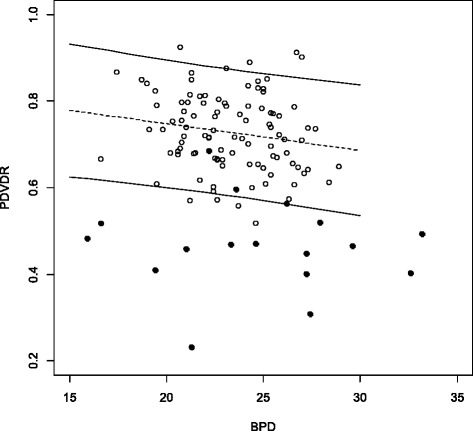
Choroid plexus diameter to lateral ventricle diameter ratio (PDVDR) in relation to biparietal diameter (BPD) in the reference group (clear circles, *n* = 100) and in fetuses with ventriculomegaly (*n* = 17, dark circles) plotted against the calculated reference range (5^th^, 50^th^ and 95^th^ percentiles)

**Fig. 4:**
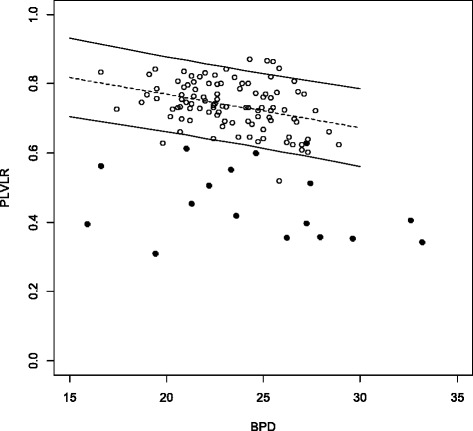
Choroid plexus length to lateral ventricle length ratio (PLVLR) in relation to biparietal diameter (BPD) in the reference group (clear circles, *n* = 100) and in fetuses with ventriculomegaly (*n* = 17, dark circles), plotted against the calculated reference range (5^th^, 50^th^ and 95^th^ percentiles)

**Fig. 5:**
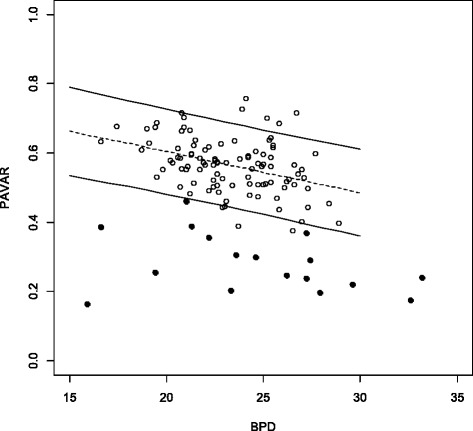
Choroid plexus area to lateral ventricle area ratio (PAVAR) in relation to biparietal diameter (BPD) in the reference group (clear circles, *n* = 100) and in fetuses with ventriculomegaly (*n* = 17, dark circles), plotted against the reference range (5^th^, 50^th^ and 95^th^ percentiles)

The PDVDR, the PLVLR and the PAVAR showed a linear decrease with increasing fetal BPD (Figs. 3, 4 and 5). The 5^th^ percentile for a BPD of 20 mm and 30 mm was 0.60 and 0.54 for PDVDR, 0.66 and 0.56 for PLVLR and 0.48 and 0.36 for PAVAR. Individual measurements from the 17 fetuses with ventriculomegaly were plotted in the calculated normal references ranges (Figs. 3, 4 and 5). Using the 5^th^ percentile as cut-off for the diagnosis of ventriculomegaly showed a sensitivity of 82.4 % for PDVDR, 94.1 % for PLVLR and 94.1 % for PAVAR, respectively. Specificities were 96.0 % for PDVDR, 98.0 % for PLVLR and 98.0 % for PAVAR. All 17 cases showed at least 2 measurements below the 5^th^ percentile, but only in 70.6 % (12/17) all three ratios were abnormal. The mean PLVLR in euploid compared to aneuploid fetuses was significantly lower (0.40 vs. 0.53, *p* < 0.05), but PAVAR (0.47 vs. 0.51, *p* = 0.41) and PDVDR (0.27 vs. 0.30, *p* = 0.43) showed no differences, respectively.

## Discussion

Although the diagnosis and causes of fetal ventriculomegaly in second and third trimester pregnancies is well described [16], little is known about early presentation at 11–14 weeks. To date a systematic approach in a screening setting is lacking and the diagnosis is usually made by the subjective evaluation of an experienced fetal medicine specialist.

The findings of our study demonstrate that in normal pregnancies the ratio between choroid plexus and lateral ventricle diameter (PDVDR), the ratio between choroid plexus and lateral ventricle length (PLVLR) and the ratio between the choroid plexus and lateral ventricle area (PAVAR) decrease with fetal BPD. The latter confirms the previous results of Loureiro et al. [10]. The observation, that in first trimester cases with ventriculomegaly there is rather a shrinkage of the choroid plexus than an increased width of the lateral ventricle, led us to the calculation of the ratio between choroid plexus and lateral ventricle length (PLVLR). In our study, this ratio and the ratio between choroid plexus and lateral ventricle area (PAVAR) were abnormal in 94 % of cases with first trimester diagnosis of ventriculomegaly. In contrast, the ratio of transverse plexus and lateral ventricle diameter showed the lowest performance for discrimination between normal and abnormal fetuses, which supports the clinical observation that the transverse diameter is less affected.

The reported prevalence of 0.2 % of first trimester ventriculomegaly in our study needs to be considered with caution, since a significant number of our patients are referred with anomalies for a second opinion and therefore the true prevalence in a screening setting is expected to be much lower. Due to the high rate of associated anomalies including aneuploidies, karyotyping should be offered in all cases. It has been shown previously, that the ratio between choroid plexus and lateral ventricular area is smaller in particular in trisomy 18 and 13 fetuses, and 32 % of fetuses with trisomy 18 and 86 % of fetuses with trisomy 13 had values below the 5^th^ percentile [10]. The incidence of aneuploidies in second trimester severe and borderline ventriculomegaly associated with structural malformations is up to 25 % [17, 18], but only 3 % in isolated borderline ventriculomegaly [14]. In our study, 31 % of those with available karyotype and more than 44 % of those with associated anomalies, presented with aneuploidies, most commonly trisomy 13. In contrast, none of the fetuses with suspected isolated ventriculomegaly and karyotyping performed, presented with a chromosomal disorder. It has been reported that in second trimester fetuses the incidence of an abnormal karyotype is lower in isolated severe compared to borderline ventriculomegaly [15]. Our study supports these findings since all 3 ratios showed lower mean values in the euploid compared to aneuploid cases even though the reported number of cases is low and therefore a statistically significant difference was only seen for PLVLR.

Counseling in the first trimester is difficult since the data on the development and progress of ventriculomegaly in the first trimester is scarce and further central nervous system (CNS)-malformations might become apparent only later in pregnancy. The reported prognosis of ventriculomegaly highly depends on the underlying cause, the ventricular width and on the presence or absence of associated conditions [15]. It may arise from a defect in brain development, may present with chromosomal defects or non-chromosomal syndromes or can be caused by hemorrhage or infection [15, 16]. In second trimester isolated forms a classification in mild (10–15 mm) and severe (>15 mm) ventriculomegaly has been shown to be useful, with a significantly different outcome in both groups. About 10 % of the children with isolated ventriculomegaly between 10–12 mm suffer from neurodevelopmental delay [19], whereas up to 50 % with severe isolated ventriculomegaly present with handicaps and only 10 % develop normally [20]. As most of our cases terminated the pregnancy in the first trimester, we can neither comment on the progress of ventriculomegaly from the first to the second trimester nor the prognosis. However, of the 5 suspected isolated cases, 2 showed CNS abnormalities during the course of pregnancy. Under the assumption that the 3 remaining cases were truly isolated, the rate of isolated ventriculomegaly is still significantly lower (17.6 %) compared to results from the second trimester (50–80 %) [18, 19, 21–23]. Another interesting observation in our study is the high association of first trimester ventriculomegaly to Dandy-Walker malformation.

Since the assessment of ventriculomegaly was based on the subjective description of specialists and the study design was retrospective, our findings need to be confirmed in further studies.

## Conclusion

The sonographic evaluation of the axial BPD plane including visualization of the butterfly sign is an integral part of the first trimester scan. We present a measurement approach for cases of suspected ventriculomegaly that can be used as a triage tool for referral to a specialist. Especially the assessment of the ratio between choroid plexus and lateral ventricle length or the ratio between the choroid plexus and lateral ventricle area were shown to be highly reproducible among different observers and could be helpful for the evaluation in the first trimester. Suspected isolated ventriculomegaly requires further investigation by fetal neurosonography in the course of pregnancy.

## Abbreviations

PDVDR: Choroid plexus to lateral ventricle diameter ratio
PLVLR: Choroid plexus to lateral ventricle length ratio
PAVAR: Choroid plexus to lateral ventricle area ratio
